# Supplementary material for: Evaluation of Prognostic Factors, including Duodenal P-Glycoprotein Expression, in Canine Chronic Enteropathy
Source: Animals (Basel). 2021 Aug 5;11(8):2315. doi: 10.3390/ani11082315 (PMC8388466; doi:10.3390/ani11082315)
Supplement: Supplementary file 1 [file animals-11-02315-s001.zip › animals-1292129-supplementary-Table S1.pdf]

N.	Gender (M: intact male; F: female; N neutered male; S: spayed female	Age (months)	Body weight (kg)	Diet previously employed (home-made 0; Commercial 1 ; Mixed diet 2)
1	M	84	35.0	0
2	M	16	35.0	0
3	S	36	3.0	2
4	F	12	28.5	1
5	M	40	45.0	2
6	F	96	23.9	1
7	S	54	7.0	1
8	M	12	33.0	1
9	M	16	31.8	1
10	N	62	6.0	1
11	M	25	41.2	0
12	F	87	29.7	1
13	F	46	18.0	1
14	M	9	6.3	1
15	M	84	27.0	2
16	M	11	25.0	1
17	M	86	46.5	2
18	M	93	11.0	2
19	M	121	18.0	2
20	M	11	27.2	1
21	M	104	34.7	2
22	M	37	7.0	1
23	F	60	15.0	2
24	M	34	30.0	2
25	F	7	18.8	0
26	M	20	22.0	2
27	M	53	53.0	1
28	M	7	25.7	2
29	F	123	4.6	1
30	M	28	24.2	1
31	F	66	41.7	1
32	F	30	7.6	1
33	S	72	22.5	1
34	M	38	38.3	1
35	M	64	20.2	1
36	F	50	28.5	1
37	M	87	33.0	1
38	M	25	42.0	0
39	M	72	36.0	2
40	M	7	26.1	1
41	M	12	45.7	0
42	M	18	12.4	0
43	S	116	14.6	2
44	F	13	23.0	1
45	M	21	36.0	2

46	M	62	11.0	0
47	M	37	6.0	1
48	S	29	6.5	0
49	M	39	31.5	2
50	M	39	7.4	2
51	M	35	17.7	0
52	M	34	9.6	1
53	M	52	5.1	1
54	S	168	11.4	2
55	M	12	12.9	1
56	S	63	4.1	1
57	M	19	6.0	2
58	M	13	25.3	2
59	M	30	18.3	1
60	M	106	33.8	1
61	M	8	39.7	2
62	M	13	63.0	0
63	M	21	31.3	1
64	M	85	9.3	1
65	F	146	26.0	1
66	M	21	10.2	2
67	M	19	27.0	1
68	M	43	25.5	1
69	S	116	7.7	0
70	M	77	9.1	2
71	S	12	26.1	2
72	M	144	9.4	1
73	M	14	28.8	1

Previous enteric parasites. protozoal and/or parvovirus infection (yes 1; no 0)	Previous treatment with Glucocorticoids (yes 1; no 0)
0	0
1	0
0	0
0	0
0	0
0	0
1	0
0	0
1	1
0	0
1	0
1	0
0	1
0	0
1	0
1	0
0	0
0	1
0	0
0	0
0	0
1	0
0	0
0	1
1	0
0	0
0	0
0	0
0	0
1	0
0	0
1	0
0	0
0	0
0	0
0	1
0	0
0	0
0	0
0	0
1	0
0	0
0	0
0	0
1	0

1	1
0	1
1	0
0	0
1	1
1	0
0	0
0	0
0	0
1	0
0	1
1	0
1	1
0	0
0	0
1	1
0	0
0	0
0	0
0	0
1	1
1	0
1	1
0	0
1	0
0	0
0	0
1	0

Decreased appetite (yes 1; no 0)	Weight loss (yes 1; no 0)	Vomiting (yes 1; no 0)	Diarrhea (yes 1; no 0)
1	1	1	0
1	1	1	1
0	0	1	1
0	1	1	0
0	0	1	1
1	1	0	1
0	0	0	1
0	0	1	1
1	1	0	1
0	0	0	1
0	1	0	1
0	0	1	0
1	1	0	1
0	0	1	1
0	0	1	1
0	1	1	1
0	1	0	1
0	1	0	1
0	0	1	1
0	1	1	1
0	0	0	1
0	0	1	1
0	1	0	1
0	1	0	1
0	1	0	1
0	0	1	1
0	0	1	1
0	0	0	1
1	1	0	1
0	1	1	1
0	1	0	1
0	0	1	0
0	0	0	1
0	1	0	1
0	0	0	1
0	1	0	1
0	0	1	1
0	1	1	1
0	0	1	1
1	1	1	1
0	1	0	1
0	0	1	0
1	1	0	1
0	0	1	1
0	0	1	1

0	0	1	1
1	1	0	1
1	1	1	1
0	0	1	1
0	0	0	0
1	1	0	1
0	0	1	1
0	0	1	1
0	1	1	1
0	0	0	1
0	0	0	1
0	1	1	1
0	1	0	1
0	0	0	1
0	1	1	1
0	0	0	1
1	0	0	1
1	1	1	1
0	1	0	1
0	0	1	1
1	0	1	1
1	1	0	1
0	1	1	1
0	0	1	1
1	0	1	0
1	1	0	1
0	1	1	1
1	0	1	1

Ascites or peripheral oedema (yes 1; no 0)	CCECAI (0-27)	PCV (%)	Platelet count (x10^3/μL)	White blood cells (x10^3/μL)
0	7	55.0	289	10.9
0	10	51.4	203	12.7
0	6	54.4	454	10.3
0	5	54.4	454	10.3
0	5	42.5	233	10.0
0	9	44.4	241	20.4
0	4	48.6	314	7.3
0	3	41.6	184	10.9
0	/	/	/	/
1	6	45.0	115	16.7
0	4	53.6	250	10.3
0	3	51.9	270	7.5
0	10	39.7	426	33.7
0	4	54.6	301	11.5
0	6	56.0	230	11.1
0	4	39.8	283	10.0
0	5	52.2	426	9.8
0	10	/	/	/
0	5	37.0	540	15.0
0	5	41.9	309	11.0
0	/	/	/	/
0	2	49.5	327	9.3
0	9	38.7	616	20.4
0	6	45.0	309	10.4
0	/	/	/	/
0	4	42.6	209	11.5
0	4	58.2	223	14.6
0	4	41.9	249	11.0
0	13	48.4	727	21.5
0	6	53.9	250	9.7
1	10	45.0	947	18.4
0	1	60.2	215	10.0
1	3	53.1	711	9.8
0	7	45.2	192	12.7
0	6	44.3	465	12.0
0	/	/	/	/
0	8	48.2	225	11.1
0	7	51.8	247	15.7
0	3	47.1	227	14.0
0	9	46.5	293	10.8
0	4	43.5	262	14.8
0	2	46.3	268	8.7
0	4	41.0	243	10.5
0	5	36.1	361	19.2
0	6	/	/	/

0	4	52.5	207	4.7
0	8	45.5	383	16.6
0	7	49.5	249	9.9
0	4	49.0	214	7.9
0	0	44.2	419	13.3
0	5	47.0	270	7.6
0	2	56.2	355	15.0
1	11	42.5	913	13.3
0	4	44.2	365	6.5
0	4	41.8	265	13.1
0	5	56.2	365	11.7
0	5	56.6	204	11.1
0	5	53.0	188	26.6
0	3	42.6	307	6.8
0	7	45.5	497	10.4
0	5	48.3	240	8.0
0	8	52.7	169	9.4
0	11	36.3	93	23.1
1	9	43.2	772	8.1
0	4	45.7	456	13.0
0	6	42.8	360	27.8
0	9	48.8	261	9.5
0	7	32.6	294	7.3
0	2	49.9	461	12.2
0	1	40.7	395	6.9
0	7	54.5	191	8.8
0	5	51.2	289	6.2
0	4	46.1	326	11.7

Serum cholesterol (mg/dL)	Serum albumin (g/dL)	Serum total protein (g/dL)	Serum cobalamin (ng/L)
220	3.74	6.30	/
256	3.11	5.81	/
218	3.38	6.18	270
218	/	6.18	270
178	3.22	5.88	210
161	1.64	3.65	/
391	3.10	3.39	417
301	3.21	5.86	/
/	/	/	/
/	1.24	2.98	433
184	3.18	6.85	264
/	3.27	6.34	/
170	2.51	5.52	666
/	3.55	/	/
190	3.27	6.67	/
146	2.77	5.52	1025
144	2.85	5.81	121
/	0.87	2.30	187
126	2.17	4.29	197
226	3.19	6.18	322
/	/	/	436
405	3.13	6.07	922
82	1.94	3.77	150
247	3.25	6.57	260
/	/	/	/
/	3.74	6.61	/
318	3.10	6.83	245
326	2.97	5.70	/
85	0.95	2.97	/
177	3.4	6.73	/
82	1.16	2.88	/
232	3.44	6.06	/
/	2.24	5.16	363
225	2.79	7.96	621
84	0.98	2.33	216
/	/	/	/
260	3.05	6.79	321
220	3.55	6.19	204
235	2.91	6.17	/
361	2.91	6.08	499
283	3.38	6.39	420
188	3.27	6.74	/
134	3.01	6.05	224
107	2.35	5.26	160
/	3.24	6.81	195

218	3.57	6.69	137
114	3.51	6.02	700
199	3.96	7.11	674
205	3.10	6.37	491
175	3.65	6.82	453
195	3.16	5.89	232
136	3.23	5.98	/
92	1.02	2.76	/
/	3.23	6.48	410
247	2.77	5.72	246
166	4.11	6.64	743
/	4.03	7.41	/
207	3.22	6.22	1000
194	2.65	5.71	314
96	1.22	3.09	156
169	3.14	6.79	/
164	3.20	6.97	297
266	2.05	6.41	/
125	1.26	2.85	/
339	3.09	7.03	/
239	3.21	7.40	/
141	2.50	5.08	165
150	1.68	3.88	466
298	3.44	7.07	/
217	2.92	6.18	663
295	3.82	6.37	/
213	3.00	5.84	468
/	3.21	6.27	427

Serum folate (μg/L)	P-gp score in *lamina propria* infiltrating lymphocytes (1-4)	P-gp score in epithelial cells (1-3)
/	2	1
/	3	2
3.10	/	/
3.10	3	1
3.39	3	1
/	4	2
9.73	3	3
/	3	2
/	2	2
1.88	/	/
4.83	3	1
/	3	1
11.28	/	/
/	1	1
/	2	1
8.23	2	1
25.00	2	1
8.54	2	2
4.77	2	1
19.9	3	1
9.31	2	1
24.00	2	1
2.28	2	1
19.30	1	2
/	1	1
/	2	1
19.30	2	2
/	2	1
/	/	/
/	2	1
/	3	2
/	2	1
24.00	2	1
10.30	/	/
24.00	2	1
/	2	2
11.12	4	2
6.20	3	2
/	4	1
12.80	4	3
4.62	4	2
/	4	2
2.82	4	2
20.00	3	2
8.14	4	3

0.93	3	2
1.70	3	3
7.15	3	2
7.34	3	3
4.09	3	3
17.91	3	3
/	2	2
/	4	2
4.81	3	1
3.79	3	1
7.26	2	2
/	2	1
10.70	3	1
12.60	2	1
17.40	3	1
/	2	2
8.27	4	2
/	/	/
/	2	1
/	/	/
/	3	1
16.00	2	1
17.20	3	2
/	3	1
17.10	2	1
/	3	1
11.60	2	1
24.00	2	1
