# Supplementary material for: Evaluation of Prognostic Factors, including Duodenal P-Glycoprotein Expression, in Canine Chronic Enteropathy
Source: Animals (Basel). 2021 Aug 5;11(8):2315. doi: 10.3390/ani11082315 (PMC8388466; doi:10.3390/ani11082315)
Supplement: Supplementary file 1 [file animals-11-02315-s001.zip › animals-1292129-supplementary-Table S2.pdf]

N.	Gender (M: intact male; F: female; N neutered male; S: spayed female	Age (months)	Body weight (kg)	Diet previously employed (home-made 0; Commercial 1 ; Mixed diet 2)
1	F	9	16.0	1
2	N	77	24.4	2
3	M	64	5.8	2
4	F	22	5.6	1
5	M	26	22.0	1
6	M	46	31.7	1
7	F	24	28.2	0
8	S	91	21.4	1
9	M	99	10.0	2
10	F	108	7.3	2
11	M	96	19.5	2
12	M	10	32.5	1
13	S	25	11.1	1
14	S	46	18.0	1
15	S	165	19.7	1
16	M	42	43.9	1
17	M	80	23.6	0
18	M	27	17.0	1
19	M	163	17.7	0

Previous enteric parasites, protozoal and/or parvovirus infection (yes 1; no 0)	Previous treatment with Glucocorticoids (yes 1; no 0)	Decreased appetite (yes 1; no 0)
0	0	1
0	1	0
0	0	1
1	0	1
0	0	1
1	0	0
1	0	0
1	1	1
0	0	0
0	1	0
0	1	1
0	0	1
0	1	0
0	1	1
0	0	0
0	1	0
0	1	1
0	1	0
0	1	1

Weight loss (yes 1; no 0)	Vomiting (yes 1; no 0)	Diarrhea (yes 1; no 0)	Ascites or peripheral oedema (yes 1; no 0)	CCECAI (0-27)
0	0	1	0	0
1	1	1	0	3
1	0	1	1	2
1	0	1	0	2
1	0	1	0	3
0	0	1	0	2
1	0	1	0	1
1	0	1	0	2
0	1	1	0	1
1	0	1	0	2
1	0	1	0	3
1	0	1	0	2
1	1	1	0	0
1	0	1	0	3
0	1	1	0	1
1	0	1	1	2
1	0	1	0	4
1	0	1	1	3
0	1	1	1	4

PCV (%)	Platelet count (x10^3/μL)	White blood cells (x10^3/μL)	Serum cholesterol (mg/dL)	Serum albumin (g/dL)
15.3	881	14.4	112	1.62
40.9	397	9.8	105	1.38
33.6	405	28.6	70	2.16
42.8	330	8.5	/	3.45
45.0	200	10.3	162	1.54
57.7	215	10.9	205	3.57
46.8	282	12.5	157	2.23
44.8	468	11.6	124	1.91
33.7	484	39.2	67	2.55
36.4	102	14.4	59	1.22
42.1	387	12.1	113	1.22
42.3	241	9.9	239	3.05
/	373	12.9	228	3.17
49.9	359	11.46	71	0.98
53.6	164	6.91	243	2.77
50.8	479	17.28	110	1.53
48.4	578	11.92	187	1.89
30.0	974	15.75	/	0.85
42.5	599	30.29	83	1.83

Serum total protein (g/dL)	Serum cobalamin (ng/L)	Serum folate (µg/L)	P-gp score in *lamina propria* infiltrating lymphocytes (1-4)	P-gp score in epithelial cells (1-3)
3.10	/	/	/	/
3.25	/	/	4	3
4.18	230	3.62	/	/
6.40	/	/	3	3
3.62	/	/	4	2
6.83	/	/	4	2
5.09	102	13.19	4	3
4.09	751	11.06	4	2
4.80	135	4.99	4	2
2.59	536	10.51	3	1
2.99	255	5.04	2	1
6.30	448	20.0	/	/
5.32	/	/	2	1
2.49	/	/	/	/
6.04	/	/	3	1
3.55	276	6.4	3	2
4.74	1000	25.0	2	1
2.23	1000	3.19	4	1
3.55	/	/	/	/
